# Statistical Modeling of Deaths from COVID-19 Influenced by Social Isolation in Latin American Countries

**DOI:** 10.4269/ajtmh.21-0217

**Published:** 2022-03-14

**Authors:** Rafael André da Silva, Luiz Philipe de Souza Ferreira, Jean Michel Rocha Sampaio Leite, Fernanda Assunção Tiraboschi, Thiago Maciel Valente, Vinicius Moraes de Paiva Roda, Jeniffer Johana Duarte Sanchez

**Affiliations:** ^1^ Life Systems Biology Graduate Program, Institute of Biomedical Sciences,University of São Paulo (ICB/USP), São Paulo, SP, Brazil;; ^2^ Biosciences Graduate Program, Intitute of Biosciences, Letters and Exact Sciences, São Paulo State University (IBILCE/UNESP), São José do Rio Preto, SP, Brazil;; ^3^ Structural and Functional Biology Graduate Program, Paulista School of Medicine, Federal University of Sao Paulo (EPM/UNIFESP), São Paulo, SP, Brazil;; ^4^ Department of Physiotherapy, University of Fortaleza (UNIFOR), Fortaleza, CE, Brazil;; ^5^ School of Public Health, University of São Paulo (FSP/USP), São Paulo, SP, Brazil;; ^6^ Department of Medicine, University of Fortaleza (UNIFOR), Fortaleza, CE, Brazil;; ^7^ Department of Statistics and Applied Math, Federal University of Ceará (UFC), Fortaleza, CE, Brazil

## Abstract

Social isolation is extremely important to minimize the effects of a pandemic. Latin American countries have similar socioeconomic characteristics and health system infrastructures. These countries face difficulties in dealing with the COVID-19 pandemic, and some of them have very high death rates. The government stringency index (GSI) of 12 Latin American countries was gathered from the Oxford COVID-19 Government Response Tracker project. The GSI is calculated by considering nine social distancing and isolation measures. Population data from the United Nations Population Fund and number-of-deaths data were collected from the dashboard of the WHO. We performed an analysis of the data collected from March through December 2020 using a mixed linear model. Peru, Brazil, Chile, Bolivia, Colombia, Argentina, and Ecuador had the highest death rates, with an increasing trend over time. Suriname, Venezuela, Uruguay, Paraguay, and Guyana had the lowest death rates, and these rates remained steady. The GSI in most countries followed the same pattern during the months analyzed. In other words, high indices at the beginning of the pandemic and lower indices in the latter months, whereas the number of deaths increased during the entire period. Almost no country kept its GSI high for a long time, especially from October to December. Time and GSI, as well as their interaction, were highly significant. As their interaction increases, the death rate decreases. In conclusion, a greater GSI at the start of the COVID-19 pandemic was associated with a decrease in the number of deaths over time in Latin American countries.

## INTRODUCTION

The COVID-19 pandemic has affected health-care systems and caused their collapse across the globe. In Latin America, the first case of severe acute respiratory syndrome coronavirus 2 (SARS-CoV-2) infection was recorded on February 25, 2020 in the city of São Paulo. In less than a month after the first case, all Latin American countries had confirmed cases of COVID-19.^1^^,^^2^

The Latin American region has several obstacles that make it difficult for countries to take action against the spread of the virus. Precarious conditions, such as poverty, lack of hospital infrastructures, subpar sanitary conditions, a high prevalence of chronic diseases, and governments’ tardy responses, are factors that make it difficult to prevent contamination by the virus, facilitate transmission, and affect hospital systems directly.^3^^–^^5^ Through predictive model studies, it has been suggested that the virus could spread aggressively throughout Latin America.^6^^,^^7^ Moreover, analyses of the initial cases of the COVID-19 pandemic in Latin America estimated an unfavorable scenario for these countries, and also evidenced aggressive dynamics in disease outbreak in Brazil and Ecuador compared with Italy and Spain.^7^ Above all, among the Latin American countries, Brazil was considered a major epicenter of the disease.^8^

Although there are measures aimed at reducing the spread of the new coronavirus, such as social distancing, school closures, cancellation of public events, and sometimes severe methods such as lockdown, these measures have been relaxed and are coupled by noncompliance by the population and poor government management.^9^ However, several current studies have already associated non-pharmaceutical interventions and lockdowns in particular with mortality, especially when measures are adopted early.^10^^,^^11^ Although recent studies have estimated that non-pharmaceutical interventions are related to the number of deaths or the rate of infection of COVID-19 in different countries,^12^ Latin American countries have not been the focus of these studies. Latin American countries should be analyzed with caution, taking into account that most of the countries in America had difficulties in facing the pandemic.^1^^,^^2^^,^^13^ In addition, Brazil is one of the countries with the highest number of deaths caused by the virus.^14^

Considering that social distancing and isolation are important protective measures for the containment of the SARS-CoV-2 infection, and that there is lack of studies examining the relationship between social isolation and death rate resulting from COVID-19 in Latin American countries, we used the government stringency index (GSI) from the Oxford Coronavirus Government Response Tracker (OxCGRT) project^15^ to analyze the relationship between the GSI and time, as well as the death rate from COVID-19 in 12 Latin American countries. We used a mixed linear model, looking at the measures adopted in the first year of the pandemic in particular. Through this model, the need for maintaining social distancing and isolation measures over time at the start of the pandemic is explained and substantiated.

## MATERIALS AND METHODS

### Data sources.

The GSI of 12 Latin American countries was developed by Oxford University and gathered from the platform Policy Responses to the Coronavirus Pandemic (https://ourworldindata.org/policy-responses-covid). This index represents the strictness of government policies and was calculated considering nine metrics of social distancing and isolation, such as school and work closures, stay-at-home requirements, transport restrictions, constraints on public gatherings, cancellation of public events, public information campaigns, restrictions on internal movements, and international travel controls. The index on any given day is calculated as the mean score of nine policy measures, each with a value between 0 and 100. Hence, a higher GSI indicates a stricter response to the pandemic. The detailed methodology for calculating indices is described elsewhere.^16^ To use these data, we first calculated the mean of the GSI for each month. The number of cumulative monthly deaths data was collected from the dashboard of the WHO. Population data were obtained from the United Nations Population Fund^17^ and were used to calculate the proportion of the number of deaths for each country. These data were collected from March 1 through December 31, 2020.

### Statistical analysis.

We evaluated the relationship between GSI and time, and the death rates from COVID-19. Because we analyzed repeated measures over a period of time (time was measured uniformly across all countries). This is a longitudinal analysis requiring a mixed linear model approach:

$$y_{ij}=\mu_{ij}+X_{ij}^{T}\beta +Z\gamma \;+\varepsilon_{\text{i}j},$$

where

$$y_{ij}=\;\frac{\text{No.}\;\text{of}\;\text{deaths}\;\text{related}\;\text{to}\;\text{COVID-19}}{\text{No.}\;\text{of}\;\text{inhabitants}\;\text{in}\;\text{the}\;\text{country}}\times 1,000,000,$$

μ is the mean of the death ratio adjusted by the total population for each 1 million inhabitants, *X_ij _*is the vector of covariates, β is the vector of the regression parameters for the covariates, *Z *is the matrix of covariates, γ is the vector of random effects, and ${\text{ε}}_{ij}$ is the vector of random errors. Gamma and ε  are uncorrelated.

In the model, the variables time (month) and GSI were both considered to be fixed. In addition, the country was included as a random effect. All statistical analysis was performed with the most commonly used significance levels (*P* = 0.01, 0.05, and 0.001) using RStudio statistical software (version 3.6; RStudio, Boston, MA).

## RESULTS

We analyzed death data related to COVID-19 in 12 Latin American countries to evaluate the relationship between death rates, GSI, and time progression. In this context, time and GSI are useful in explaining the dispersion of the data.

**Figure 1.**
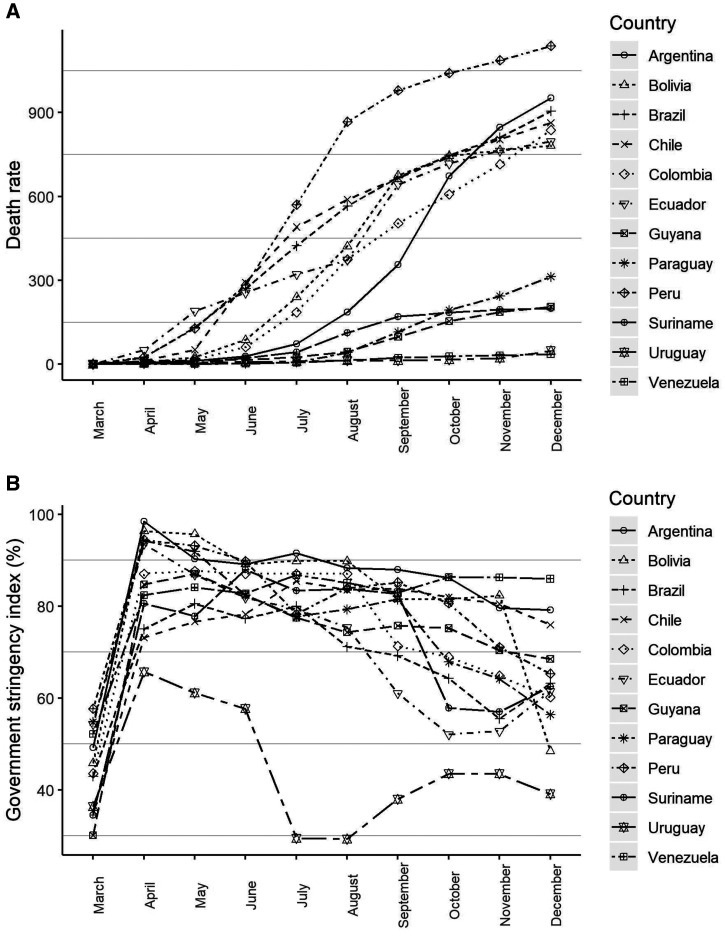
Death rates from COVID-19 and the government stringency index from March to December 2020 in Latin American countries. (**A**) Death rates adjusted by population size for each country per million inhabitants. (**B**) Mean government stringency index reported as a percentage.

Figure 1A shows death rates from March to December. Peru, Brazil, Chile, Bolivia, Colombia, Argentina, and Ecuador have high death rates, with an increasing trend over time, whereas Suriname, Venezuela, Uruguay, Paraguay, and Guyana have low death rates, which remained stable. Figure 1B shows the GSIs from March to December. It is noticeable that there was much fluctuation in the GSI for most countries, but with a large decrease from October to December. The only country with a GSI that remained high for the entire period was Venezuela.

Also, in Figure 1A, it is possible to observe the asymmetry of the data, so a skewed *t *distribution was adopted for the model error. Because the model presents a variable dispersion, we used a linear regression model for the dispersion:

$$\begin{array}{l} y_{ij}|{\text{time}}_{i},{\text{country}}_{j},{\text{GSI}}_{ij}∼ST(\mu_{ij},\sigma_{ij},\nu ,\tau ) \end{array}$$

$$\begin{array}{l} \mu_{ij}^{-1}=\frac{1}{\mu_{ij}}=\beta_{0}\;+\beta_{1}\;{\text{time}}_{i}+\beta_{2}\;{\text{GSI}}_{ij}+\beta_{3}\;{\text{time}}_{i}\times {\text{GSI}}_{ij}+\gamma_{0}+\gamma_{1}{\text{country}}_{j}\\ log\left(\sigma_{ij}\right)=\alpha_{0}+\alpha_{1}{\text{time}}_{i}+\alpha_{2\;}{\text{GSI}}_{ij}, \end{array}$$

where *i *= 1, … 10 represents each of the months, starting from March; *j *= 1, … 12 corresponds to each of the countries; time*_i_* represents the *i*th month, country*_j_* represents the *j*th country, GSI*_ij_* represents the GSI in the *i*th month in the *j*th country, and σ*_ij_* corresponds to the SD in the *i*th month in the *j*th country, with its corresponding parameters α. The random effects γ_0 _and γ_1_ have a normal distribution, and the response variable has a skewed *t* distribution, with parameters μ*_ij_*, σ*_ij_*, ν, and τ.

Table 1 shows the coefficient estimates of the mixed linear model and the dispersion model. The random effects are random variables that do not assume a single value, which means that its values cannot be displayed. Time and GSI, as well as their interaction, were highly significant to explain the death rate under all assumed significance levels. As the interaction of time and GSI increases, the death rate decreases.

**Table 1:** Estimates of the dispersion model and mixed linear model for death rates from COVID-19 in 2020 in Latin American countries

	Estimate	SE	*t* Value	*P *value
Intercept, β_0_ (μ)	491,633.0	69,250.3	7.099	< 0.005***
Time, β_1_ (μ)	–70,118.5	12,794.0	–5.81	< 0.005***
GSI, β_2_ (μ)	–6,721.4	984.6	–6.826	< 0.005***
Time × GSI, β_3_ (μ)	953.6	189.2	5.041	< 0.005***
Intercept, α_0_ (σ)	–3.89	0.59	–6.63	< 0.005***
Time, α_1_ (σ)	0.72	0.04	16.67	< 0.005***
GSI, α_2_ (σ)	0.05	0.01	8.00	< 0.005***

GSI = government stringency index; μ = mean of the death ratio adjusted by total population for each million inhabitants; SE = standard error; σ = dispersion.

The relationship between the predictors and the original response variable is inversely proportional. In other words, a negative sign indicates an increase of death rates whereas a positive sign indicates a decrease. Importantly, considering the interaction effect was significant, the main effects cannot be interpreted individually. For instance, the interpretation from the estimate obtained for the parameter associated with the interaction (953.6) is that for a given fixed month; if the GSI increases, the death rate decreases.

*** Significant at *P *= 0.001.

In Supplemental Figure S1A, the QQ-plot envelope shows there is no evidence that the skewed *t* distribution is inappropriate to explain the death rate for each million inhabitants. Other aspects of the model were analyzed using quantile residuals (Supplemental Figure S1B), such as the correct specification of the model’s dispersion and distribution. We can conclude from these graphs that the model satisfies the assumptions, so the model specification is appropriate.

## DISCUSSION

Robust evidence shows that, under most conditions, early adoption of stringent government non-pharmaceutical interventions is associated with a reduction in transmission and death.^10^^–^^12^ Continued intervention should be considered to keep transmission of disease under control.^10^ In addition, it was estimated that the GSI was able to decrease the number of deaths at different waves during the course of SARS-CoV-2 disease in different countries.^12^ However, addressing the influence of this factor on death rates remains a big challenge, because countries publish their testing data at different times. Some provide daily updates whereas others provide them on a weekly-basis, and yet others publish figures on an ad hoc basis at longer intervals.

Based on the GSI data extracted from the OxCGRT project,^15^ it is possible to propose statistical models to evaluate how closely these variables are related to time. Our model shows that the relationship between time and GSI is highly significant. When analyzing time and the GSI together, it was observed that, as the interaction of these two variables increases, a decrease in the death rate is detected. For instance, according to this model, with a GSI set to 0 in March and a GSI set to 80 in April (i.e., an 80% increase in the strictness of government policies), we would observe a reduction of approximately 32 deaths/1 million inhabitants. In Brazil, with a population close to 212 million, this represents 6,784 lives that could have been saved at the beginning of the pandemic. This figure is even higher in other months within the analyzed period. Furthermore, when these two variables were analyzed separately, as time increases, the rate of deaths per 1 million inhabitants increases as well. Surprisingly, the same happens with the GSI reported by the countries. In light of these observations, we can make two hypotheses. First, the GSI alone may not entirely represent the reality regarding social isolation and the death rate from COVID-19, because this condition depends on other factors, such as the infrastructure of the countries’ public hospitals, government management, and the population’s compliance with the rules. Second, restriction policies as measured by the GSI do not have immediate effects and must be maintained over longer periods to decrease death rates by COVID-19. Hence, the problem is complex and deserves to be studied in detail, taking into account other aspects that may be influencing the death rates.

Latin American countries present problematic issues, such as social inequality and less access to health care. In addition, complying with social isolation is difficult for individuals when work is the only source of income.^13^ In our analyses, countries such as Peru, Brazil, Chile, Bolivia, Colombia, Argentina, and Ecuador have the highest death rates. Peru has inadequate conditions to face a pandemic, and even in lockdown at the beginning of the pandemic,^2^ it presented high death rates. In a prediction study with data from the first 10 days of the pandemic, it was estimated that Peru had the lowest effective reproductive number (R*_t_*), a parameter used to keep track of epidemics.^7^ Therefore, the country had these numbers accentuated during the pandemic period.

Brazil was the first Latin American country to report cases of COVID-19.^18^^–^^20^ Because it has favorable conditions for facing a pandemic compared with other Latin American countries, it was expected to have lower rates. However, in our study, Brazil and Chile had higher death rates, followed by Peru. It is important to consider that, although Peru’s president has played a relevant role in helping to control the number of deaths from COVID-19, there has been neither a national strategic plan to guide communication and educational health policies nor a large-scale awareness campaign to stimulate people to protect their health and adhere to protective measures. This lack of policy is also a current problem in Brazil.^2^ For instance, through the GSI and COVID-19 Community Mobility Reports from Google, daily new cases and real-time R*_t_* values were calculated that show that Brazil is not doing very well regarding its response to the COVID-19 pandemic.^21^ Although Brazil presents a robust public health system and a reasonable GSI, the high death rates may be deeply connected to inadequate policy management, which has been criticized.^5^^,^^13^ In comparison to Brazil, Suriname had a similar GSI but a low death rate that remained stable over time. Except for Venezuela, no other country kept a high GSI for longer periods. In particular, the GSI decreased at the end of the year (October–December) in most countries, whereas death rates increased. On the other hand, isolation in Venezuela was maintained even in December (an atypical month because of the holiday season), and its death rates were low and remained unchanged over time.

According to our analysis, Uruguay had a relatively lower GSI than other Latin American countries, but demonstrated low death rates. Uruguay is a country that acted quickly to the pandemic, closing its borders and schools, promoting screening tests, reducing SARS-CoV-2 infection, and controlling the outbreak very efficiently.^22^ In contrast, Ecuador started with high social isolation, but a decrease in the isolation rate was observed later. On the other hand, Ecuador had a high mortality rate, which is accentuated over time, even with the adoption of lockdown. In addition, it should be noted that Ecuador had a poor public health infrastructure at the beginning of the pandemic.^2^ At the beginning of the COVID-19 pandemic, it was suggested that discontinuing public transportation, and closing workplaces and schools were particularly effective in reducing COVID-19 transmission.^23^

The rapidly evolving pandemic in Latin American countries is worthy of special attention, considering their often weak and low stringency responses to the current sanitary crisis. In our study, the GSI varies considerably in all Latin American countries over time. This variation can explain in part why these countries have been affected differently by COVID-19. Despite not specifically addressing and discussing the government policies adopted by each country, in this investigation, we successfully show that social distancing and isolation measured by the GSI influences death rates from COVID-19 over time. For instance, the interaction between the GSI and time can decrease the number of deaths, which demonstrates the importance of maintaining social distancing and isolation measures for longer periods, as opposed to what most Latin American countries did. Almost no country kept its GSI high for long, especially from October to December. We did not expect to find different results, because several studies support the idea that strict policies are extremely important to contain the contagion or death from COVID-19.^10^^–^^12^ This is the first article that discusses the importance of non-pharmaceutical interventions based on increased GSIs that could have directly affected the number of deaths in Latin American countries.

Our results have significant implications; however, some limiting aspects must be considered. First, the GSIs were extracted from the OxCGRT project. The curators of this database emphasized how challenging the collection of information on the exact data was because of the nature and extent of the policies of the different governments. This complex data set can obscure qualitative differences in each of the nine metrics the GSI measures across countries. In addition, many local and cultural factors can affect the implementation of interventions. Second, our data provide a general interpretation of the influence of time and the GSI on death rates in Latin America. Therefore, future studies can deepen the search for more specific interpretations for each country, taking into account local aspects and other metrics not covered here. Third, the numbers of deaths from COVID-19 can be easily underreported^20^^,^^21^ as a result of limited testing, problems in determining the cause of death, and the way in which COVID-19 deaths are recorded. Hence, we cannot define the real impact of the GSI on death rates with precision. Fourth, we know that the difference in population size among countries is often large, and the COVID-19 death count in more populous countries tends to be greater. Thus, to perform a more truthful comparison, we used cumulative death data and calculated the death rate adjusted by the population of each country.

## CONCLUSION

We conclude that, in combination, time and the GSI have beneficial effects on the decrease of death rates from COVID-19 in Latin American countries. Greater strictness regarding social distancing and isolation, as measured by the GSI, at the start of the pandemic, could have flattened mortality curves from COVID-19 over time, particularly from March to December 2020. Our statistical model explains and substantiates the need for maintaining social distancing and isolation measures over time during the pandemic.

## Supplemental Material


Supplemental materials
