# Supplementary material for: Statistical Modeling of Deaths from COVID-19 Influenced by Social Isolation in Latin American Countries
Source: Am J Trop Med Hyg. 2022 Mar 14;106(5):1486–90. doi: 10.4269/ajtmh.21-0217 (PMC9128698; doi:10.4269/ajtmh.21-0217)

Supplementary Materials: Analysis of the model fit

Supplemental Figure S1A) Q-Q plot envelope. Supplemental Figure S1B) Residual analysis.

S1A)

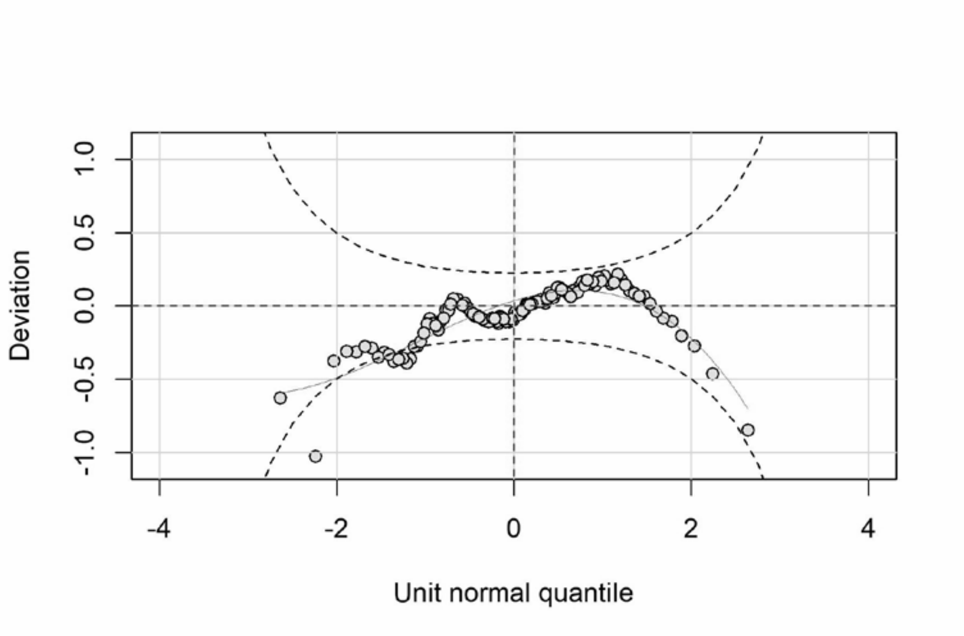

S1B)

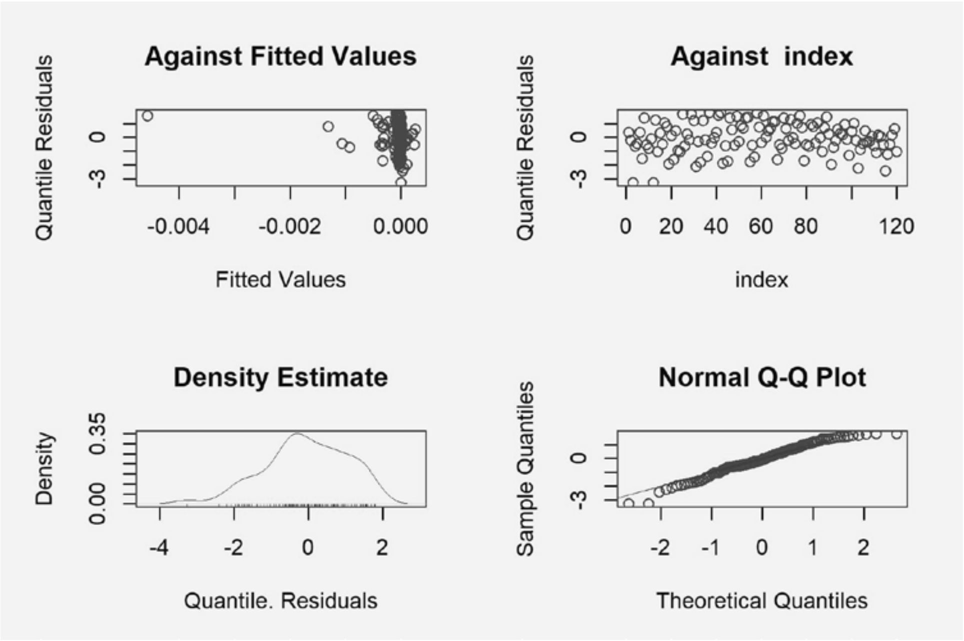

Supplement: Supplementary file 1 [file tpmd210217.SD1.pdf]
